# The Physician’s Leisure; a Plea for the Study of Archæology

**Published:** 1881-06

**Authors:** 


THE PHYSICIAN'S LEISURE; A PLEA FOR THE STUDY OF ARCHÆOLOGY. By Dr. CHEVERS. (Continued from p. 548, May, 1881.)

The ancients had their air-tight dressings, ruder than the appliances of the antiseptic method, but useful in the same direction. Wadd tells us of an empiric who professed that he had a radical cure for inguinal hernia. He used to remove the testicle and throw it to his dog, and then to secure the cord with a sow-gelder's clamp in such a manner as, possibly, to set up inflammation in the inguinal canal, and to close it against the descent of the hernia, providing the patient did not die. This wretch rudely anticipated an operation which modern science has almost perfected. In John Hunter's time and long afterwards, broad ligatures or narrow tapes were used in tying arteries. I had the honor of knowing, in his retirement at Kensington, Dr. Veitch, a naval surgeon, who first proposed the thin round silk ligature—certainly one of the most daring and remarkable discoveries ever made in surgery. "How," common-place people must have exclaimed, "is a great artery to be closed by suddenly cutting through its inner and middle coats?" And so it has been that, for the last seventy years, no one has amputated a limb or deligated a main artery without dreading hæmorrhage should the ligature separate too early. Now again, Mr. Barwell has introduced a most ingenious modification of the broad ligature. His "flat ligature," which compresses the artery but does not divide its coats, has been employed with great success.

The palæontologist and anthropologist search with true antiquarian feeling, in the caverns and pits of Britain, for the remains of the bear, the wolf, the wild-boar, the hippopotamus, and rhinoceros, and delve in the barrows which stud our English downs for the skulls and weapons, and ornaments of our brachy-cephalic and dolicho-cephalic ancestors. Others explore the mosses of Waterford and the Isle of Man for the skeletons of the extinct gigantic elk (*Magaceros Hibernicus*), and the icebergs of Spitzbergen for the frozen bodies of the mammoths of the glacial period. At home we view with interest the *Bos primigenius*, the only pure, but degenerate, representative of the aboriginal gigantic cattle of our island, still to be found at Hamilton, Chillingham, and Chartley. When duty compels us to labor in distant colonies, we reconstruct the bird dodo of the Mauritius, and the dinornis and apteryx of New Zealand, and we discover living types of the fossil iguanodons and crocodilians of the oolite in the go-samp of the East and the guano of the West Indies, and in the garials, representing the genera Steneosaurus and Teliosaurus, at which griffs throw pot-shots, usually ineffectually, as the boats stem up against the tide of the Ganges and Brahmapootra. The zoologist observes that the edible snail (*Helix pomatia*), cultivated by the Roman colonists in Britain more than nine centuries ago, as freely as snails are eaten in Paris at this moment, is still so often to be found in the vicinity of Roman settlements, that its discovery in any given locality encourages the antiquary to search its habitat for the buried remains of Roman mansions.

So the botanist wonders over the mummy wheat and the mummy pea of Egypt, preserved in life among the cerements of the embalmed dead since the time of the exodus. He notices a fact of similar interest in this passage from Aubrey's Natural History of Wiltshire: "The spring after the conflagration at London, all the ruins were overgrown with an herbe or two, but especially one with a yellow flower; and on the south side of St. Paul's Church it grew as thick as could be; nay, on the very top of the tower. The herbalists call it *Ericolevis Neapolitana*, small bank cresses of Naples; which plant Tho. Willis" [the anatomist and physician, from whom the circle of Willis has its designation] "told me he knew before but in one place about the town; and that was at Battle Bridge, by the Pindar of Wakefield, and that in no great quantity." Again the same conservative spirit in

Nature, in now hoarding up and now spreading abroad buried germs of useful plants for man's benefit, is illustrated by a fact mentioned to me by my friend Sir Joseph Fayrer, and lately cited in My Notes on the Diseases of India, that, after the incessant cannonade of the enemy had crumbled to dust much of the frail defenses which surrounded the Residency at Lucknow, the wild spinach of India (*sag*), a useful antiscorbutic, sprang up from the *débris* of pulverized brickwork, and was brought in by our soldiers, under fire, and sold, weight for weight against rupees, to the officers, who bought it for their wives and children perishing from scurvy. We were told, a few years ago, that red roses still flourish wildly where so much Lancastrian blood was poured out four hundred years ago, on the field of Towton. The Scottish thistle, doubtless planted by the attendants of Mary Stuart, still flourishes on the site of Fotheringhay, although not one stone of that great ducal fortalice remains upon another; and it is a well-known fact, in the North, that the line of the Roman wall is peculiarly rich in those medicinal plants which were used by the Roman physicians.

The geologist, the botanist, and the zoologist naturally and readily become antiquaries. Their work requires that they should visit the remotest and least frequented districts; and, while they examine the structure of the rocks or the flora and fauna of each locality, they readily observe and perhaps discover antiquities which time has hidden from eyes less practiced. Thus it is that, in our own time, Nilson, the zoölogist, has taken a foremost place among northern archæologists.

The chemist must have a very unlearned knowledge of his science if he cannot give the history of his predecessors, the alchemists,—Raimond Lully, Basil Valentine, who discovered the medical uses of antimony, and Paracelsus; and unless he can show how chemistry was developed by Boyle, Cavendish, Lavoisier, Priestly, and Davy.

Many medical men, like Charles Patin, the numismatist, Sir Thomas Brown, Dr. William Stukeley, and Professor Simpson, whose memory is still fresh wherever medicine is valued in its highest scientific aspects, were eminent antiquaries—that faculty which enables a man to discover how to read the inscription on a coin which time has rendered, to all appearance, perfectly flat, by placing it on a plate of red-hot metal, being nearly akin to that power of cultivated observation by which the mysteries of pathological action are investigated and interpreted. Chief among modern archæologists is to be named a gentleman long a general practitioner in the city, in the center of the remains of Roman London. Encouraging the mud-larks of the river Thames, and the laborers who dug the sewers and laid down the gas mains, he gradually collected from the river shingle, from the bed of the ancient Wall-Brook, by the Mansion House, and from the dust pits of the city of the Proconsuls, so rich a store of Samian ware, such a variety of ornaments, so many works of art, statuary, tesselated pavements, sarcophagi, utensils, and weapons, that Roman London was virtually re-discovered by his researches, and lives again, at the Guildhall, after fifteen centuries of obliteration, in the collections of Charles Roach Smith.

What a solace may archæology become to the perhaps not extremely successful country doctor who has within him a scintilla of real antiquarian feeling! His daily round, perhaps through mile after mile of the bleakest, barest, ugliest country in the realm, becomes to him a course of never failing interest. We will say that it is nearly all down or moorland, without a grove or a single stately tree to afford it shade or to relieve its blankness, or one babbling stream or pleasant waterfall to give it life and freshness. And yet how full of interest it is to him! Yonder hideous church tower is nearly unique as an unrestored specimen of well authenticated Saxon architecture. In the churchyard is a rude stone cross, the inscription on which he has been enabled to read by aid of the Runic alphabet in his Penny Cyclopædia. The stump on this knoll by the wayside shows where Hardriding Rob, the highwayman, was gibbeted in chains. That shapeless mass of coarse stone rubble, traversed with bright red sections of baked tiles, is one of the *vestigia* of a Roman city which lies buried here, and from the confines of which the country folk often bring him relics, of which he alone, in that neighborhood, knows the true import and historic value, ranging from the invasion of Claudius and throughout the four hundred years of Roman occupation until the last trireme left our shores. From this old chalk-pit the diggers (he is not to be imposed upon by the Stone Jacks who manufacture these relics) procure him and sell him for copper those palæolithic and neolithic flint spear heads, arrow heads, and hatchets, his collection of which is beginning to obtain celebrity. Across that valley is a British earthwork, and upon it the still distinctly bared cutting of a gigantic human figure, most clearly defined when rain has fallen and the tender grass springs up along its outline. In this, enclosed within strong hurdles, the Druids used to sacrifice human captives by the hecatomb. Out there are Celtic barrows in all their varieties of form, the long and the conical, the twin barrow and the Druid barrow, the burial places of prehistoric chieftains. Just here was fought one of the grimmest battles of the Wars of the Roses. See how bravely the corn waves where the dead lie thickest, and where, as lately happened in the "Bloody Meadow" battle-field at Tewkesbury, the teeth of the flower of England are brought to light wherever the plowshare passes. On the green sward, a little farther on, once a Roman *cursus*, one of Rupert's fiery charges broke a squadron of ironsides as a gust of east wind scatters the thistle-down.

The museum of the nearest city, so much indebted as it is to his knowledge and contributions, is, to him, a place of delightful resort. The sight of a new number of *Notes and Queries*, containing his reply to a long debated question, upon his study table when he returns at night, almost worn out by toil, to which disappointment has perhaps added tenfold weariness, seems to renew the life within him; and the occasional festival of the Archæological Society, of which he is an active member and the chief local cicerone and expositor, affords him unalloyed delight.

Some matter-of-fact hearers may say—"All this is very well, by way of amusement, to the few who care for such old-world matters; but you have described to us a mere unpractical Dry-as-Dust who wastes his time and his money in desultory ramblings and earth grubbings which bring him and his patients no valid and substantially useful return. It is not so. His leisure is pleasantly and, at the very least, harmlessly occupied; utter weariness of the toils and troubles of life is not permitted to eat his soul up; and, for the rest, the people respect him for his great earnestness, his strange knowledge, and his pure life; and his intercourse with the clergy and squirearchy of his district is rendered safe and pleasant by their recognition of the fact that their medical attendant, whose professional knowledge they have no power of gauging, is undoubtedly a gentleman of refined and scholarly tastes. Let me repeat—Your patients have, for the most part, very little notion as to whether you are or are not competent physicians and surgeons. In this respect any impudent quack will often strike and attract the laity more than you will ever succeed in doing. But you will fail in your duty to yourselves and your profession if you do not take honest, unostentatious means to acquaint the society into which you are thrown that you are well educated men of cultivated intelligence.

Here I must offer a caution which, with some, will condemn my main argument. However praiseworthy and useful the occupations of the physician's leisure may be in themselves, he must pursue them with some caution, I will not say secresy, at least until his professional reputation is fully established. The enemy readily discover a weak point; any gibe that stings a rival will serve in turn. It was said of a student who went in for the prize in medicine, "We must either give the medal to him or give him nothing." "Well, he shall have the latter,—he is a *bookish* man." I knew a surgeon who fell very little short of greatness, who failed in life because he had become branded with the name of "pathologist." It must not be charged against us that we sacrifice our duty to our amusements or even to our special studies. "Dr. ——," said a patient, "is probably too much engaged in his literary pursuits to afford proper attention to the sick." John Bell's remark upon his brother and superior, Sir Charles, is alleged to have been, "The body draws." I was present when it was proposed, but not seconded, in our municipality at ——, that the city surveyor, an exemplary officer, but an admirable amateur photographer, should be ordered to give over taking sun-pictures. But this difficulty attends nearly all visible and active amusements, and he is unfit for our profession who is not both discreet and moderate.

To myself, a strong, but, I trust, not ill-regulated, love for antiquarian reading has (to say little of the rest and gratification which it has given in an active career in which ordinary "amusement" has obtained no part at all) afforded me great and valid assistance as a professor of medicine, as a hospital physician, and as an investigator and writer in the subjects of pathology, public hygiene, and medical jurisprudence, for research in which India affords an admirable field. In all my subjects of study, old books yielded me endless lessons. Let us take one of these. Throughout that web which forms the etiology of most of the prevailing diseases, not only of India, but also of England, run two threads of pathological influence, by the accurate tracing of which the physician can often alone hope to unravel the mesh in which his patients are enveloped. These two factors are the marsh poison and scorbutus. What modern physician, of English training, can efficiently cope with these most insidious and tenacious constitutional taints unless he has paid close attention to history? He can have seen very little pure and unmasked malarious fever in the city hospital where he studied; but, at this moment, there are plenty of marshes and bad food in the United Kingdom; and history tells him that, in ancient times, marsh disease swept down the haughtiest heads of England's royalty and nobility. Henry of Agincourt, Wolsey, Walter Devereux, Lord Deputy of Ireland, and a host of other distinguished persons, died of dysentery or some cognate disease. Mary of England, Cardinal Pole, James the First, Cromwell, and Charles the Second died of marsh fever. In fact, it is a truth, albeit a quaint one, that the Reformation was, validly, the first great step in the march of sanitary reform in England. It led to the filling up of the moats and stews, fish ponds and lakes, which furnished diet for fast-days, and which maintained a constant supply of paludal poison at the very door of every country house in England, as the tank or water pit now does beside each hovel in Bengal. Again, who, although he may have worked for years in a seaman's hospital, either in this country or in India, can know much of fully developed scurvy unless he has pondered over the miserable but invaluable lesson afforded by the narrative of Anson's voyage, and has made himself thoroughly acquainted with sea scurvey as its every phase is delineated in the now antiquated pages of Lind and Trotter? Land scurvy is present nearly everywhere among the poor, and often among the rich, in India; and it and the marsh poison should never be entirely out of the mind of the physician who practices in London, one of the worst placed cities in the world, where the combined effects of these two deadly influences are daily seen in purpura, anæmia, sloughing phagedæna, cancrum oris, carbuncle, dysentery, and splenic enlargement.

Still again, who can take a large view of diphtheria, typhus, and the true enteric fever of Jenner, unless he knows well the history of their great predecessor, the Plague, which appeared nearly every year in filthy, overcrowded, ill drained London, and heaped up fathoms of dead in the monstrous plague pits (one of which lies within a few paces of the spot upon which we now stand), until that grand but rather costly sanitary measure, the Great Fire of 1666, only improved matters in degree, leaving in the Plague's track the scarcely less insidious and mortal diseases which I have just named; and also, as I fully believe, still leaving this improved but very insanitary city fully open to attacks of plague whenever the pestilential wave shall again be directed northwards?

Who could, with advantage to the miserable individuals under his charge, work, either in England or as I did in India, as an inspector of jails, unless he knows the history of the Jail Fever in England, which called into activity Howard and his noble system of prison reform. How, as Stow tells us, in 1414, "the Galores of Newgate and Ludgate dyed, and prisoners in Newgate to the number of sixty-four—through a contagion called the Sickness of the house;" how, in July, 1577, at the Black Assize, at Oxford, those in court were surprised with a pestilent savor, upon which the Lord Chief Baron, almost all the jurors, and three hundred others who were in the court, sickened and died; and how, in May, 1750,—a range, not an intermission, of nearly three hundred years,—at the session of the Old Bailey, four on the bench together with two or three of the counsel, one of the under sheriffs, several of the Middlesex jury, and others present to the amount of forty-nine, were attacked with the disease and perished?

To the working student of public hygiene, a true and full knowledge of the history and minute topography of Old London before the Fire, when a hundred and thirty thousand citizens were packed, almost in bulk, into the narrow walled space between Cripplegate and Queenhithe, the Fleet River and Aldgate, is what a hospital and a dead-house are to the practical physician. In the records of the one will be found the most typical illustrations of nearly all insanitary things in their full, unchecked development, as in the beds and on the tables of the others will be discovered all morbid objects, open to him who can observe. If a student of medicine tells me that he has just seen two "good cases," I, as a student of old-world sanitation, can give him two, out of hundreds of equal interst. 1st. I read* that, on October 9, 1551, Giles, the King's beer brewer, dwelling at St. Catherine's, *who had bled to death from a scratch on his leg*, was buried this day at Aldgate, with heraldic emblazonments of his arms and the craft of the brewers. Here is a very natural death (of which more than one parallel instance has come under my notice) in a person who breathes a mixture of marsh and sewer gases, and has unrestrained access to intoxicating liquor. 2nd. I note that the inhabitants of the houses on London bridge always escaped the Plague—an early and very striking evidence of the value of free sewage and thorough perflation.

It is a remarkable, but by no means new, observation, that, when I have been engaged in search of any particular branch of knowledge, I have scarcely ever taken up the most ancient and best of all books without discovering, to my surprise, information closely germane to my inquiry. I will give only two instances out of many. The tanks of Bengal are ponds upon which the sun generally shines all day long, and the natives are constantly seen bathing or drawing water there. Magistrates and civil surgeons are well aware that a by no means unhealthy looking Bengali is not unfrequently found lying dead, face downwards, in very shallow water. On post mortem examination no traces of mortal disease are discovered, and much doubt has often arisen as to how the individual came by his death. The neighbors generally say that he had *mrigi*, epilepsy; and this is probably true in many cases. Then there are also many other instances in which Indian epileptics fall into the fire or into the huge kettles

* Henry Machin's Diary.

in which rice is boiled for large parties at festivals. When I mentioned this to Dr. Francis Warner, he remarked that he had recently seen a case in which the epileptic paroxysm was excited by the sight of any bright or dazzling object. So it always is with the spasm of hydrophobia. How clearly is this law in disease expressed in the sacred account of the epileptic demoniac: "Ofttimes it" [the dumb spirit] "hath cast him into the fire, and into the waters, to destroy him" (Mark ix. 22).

Again, it is well known that the Cucurbits are a rather dangerous family, the worst of their number being *Cucumis colocynthis;* but gourds, melons, and cucumbers are generally considered to have a doubtful character for wholesomeness. In working out the subject of Indian poisons, I of course inquired about the Cucurbitaceæ, but found little or nothing for the three editions of my book except the case of a retired officer, at a hill station, who was attacked with symptoms of irritant poisoning, and rapidly died, after eating kuddo, a generally inoxious pumpkin. In May, 1871, there appeared in the *Indian Medical Gazette* two cases of poisoning by *Cucurbita lagenaria*, a wild variety of white pumpkin or bitter cucumber. Not long after I had made a note of this, I came unexpectedly upon the passage * in which we are told that Elisha prepared to scethe pottage for the sons of the prophets, "And one went out into the field to gather herbs, and found a wild vine, and gathered thereof wild gourds his lap full, and came and shred them into the pot of pottage: for they knew them not. So they poured them out for the men to eat. And it came to pass, as they were eating the pottage, that they cried out and said, O thou man of God, there is death in the pot." Here were two absolutely new cases of the present day, standing quite alone and needing confirmation, distinctly capped and paralleled by the very oldest case of poisoning on record!

To the occupations which I have recommended we may justly apply the words of Cicero;—"Hæc studia adolescentiam alunt, senectutem oblectant; secundas res ornant, adversis perfugium ac solatium præbent; delectant domi, non impediunt foris, pernoctant nobiscum pereginantur, rusticantur."

These remarks are not intended for medical men exclusively.

* 2 Kings IV, 38.

In their general drift they have application to men of all professions who live by a combination of physical toil with mental effort.

"Cease to work on Sundays," was Johnson's dying adjuration to Joshua Reynolds. Medical men are, of course, unable to command any given moment of their time; still, all those who wish it can generally make Sunday a day of rest. It is clearly indispensable that the medical man shall be an early riser, and, when in large practice, he is rarely allowed to waste time in sleep; but, whenever he can get it, his sleep should be quite *ad libitum.* I could never understand a judge who boasted that he restricted himself to five hours' sleep; but it appeared to me that the singular irritability and want of dignity and equanimity which he displayed on the bench were largely attributable to this cause. The greatest and most laborious ruler of India in modern times, Lord Dalhousie, considered that hard study or intense mental labor of any kind cannot be habitually maintained longer than six hours. When more is attempted, the result is muddle. Of one practical fact there can be no doubt: he who works and thinks hard all day cannot afford, systematically or frequently, to spend half the night in any frivolous amusement or intense study. Many elderly men lose their reserve force and break down in vainly attempting these modes of life.

I ought perhaps to apologize here to those who have listened to these details with attention, which is, at once, so courteous and so kind, considering that they, admittedly, have no taste for antiquarian pursuits. Let me entreat them to recur to the advice conveyed in the first part of this address. Let them allow themselves, throughout their active life, a fair amount of rest and recuperative leisure, and search diligently for some well chosen and appropriate mode of occupying that leasure, and employ it, as they work out their professional duties, in a manner becoming the noble calling of enlightened scholars, gentlemen, and physicians.

